# Randomized Online Computation with High Probability Guarantees

**DOI:** 10.1007/s00453-022-00925-z

**Published:** 2022-01-30

**Authors:** Dennis Komm, Rastislav Královič, Richard Královič, Tobias Mömke

**Affiliations:** 1 Department of Computer Science, ETH Zurich, Zurich, Switzerland; 2 Comenius University, Bratislava, Slovakia; 3 University of Augsburg, Augsburg, Germany

**Keywords:** Online algorithms, Randomization, Bounds with high probability

## Abstract

We study the relationship between the competitive ratio and the tail distribution of randomized online problems. To this end, we identify a broad class of online problems for which the existence of a randomized online algorithm with constant expected competitive ratio *r* implies the existence of a randomized online algorithm that has a competitive ratio of $(1+\varepsilon )r$ *with high probability*, measured with respect to the optimal profit or cost, respectively. The class of problems includes some of the well-studied online problems such as paging, *k*-server, and metrical task systems on finite metric spaces.

## Introduction

In online computation, we face the challenge of designing algorithms that work in environments where parts of the input are unknown while parts of the output already need to be provided. The standard way of evaluating the quality of online algorithms is by means of *competitive analysis*, where one compares the outcome of an online algorithm to the optimal solution constructed by a hypothetical optimal offline algorithm. Since deterministic strategies are often proven to fail for the most prominent problems, randomization is used as a powerful tool to construct high-quality algorithms that outperform their deterministic counterparts. These algorithms base their computations on the outcome of a random source; for a detailed introduction to online problems we refer the reader to the literature [3, 9].

The most common way to measure the performance of randomized algorithms is to analyze the worst-case expected outcome and to compare it to the optimal offline solution. With offline algorithms, a statement about the expected outcome is also a statement about the outcome *with high probability* due to Markov’s inequality and the fact that the algorithm may be executed many times to amplify the probability of success [7]. This amplification, however, is not possible in online settings. As online algorithms only have one attempt to compute a reasonably good result, a statement with respect to the expected value of their competitive ratio may be rather unsatisfactory. As a matter of fact, for a fixed input, it might be the case that such an algorithm produces high quality results in very few cases (i.e., for a rather small number of random choices), but is unacceptably bad for the majority of random computations. Nevertheless, the expected competitive ratio might suggest a better performance. Thus, if we want to have a guarantee that some randomized online algorithm obtains a particular quality with some given probability, we must have a closer look at its analysis. In such a setting, we would like to state that the algorithm does not only perform well on average, but “almost always.”

Besides a theoretical formalization of the above statement, the main contribution of this paper is to show that, for a broad class of problems, the existence of a randomized online algorithm that performs well in expectation immediately implies the existence of a randomized online algorithm that is virtually as good with high probability.

Our investigations, however, need to be detailed in order to face the particularities of the framework. Specifically, our goal is to establish results with high probability that are independent of the concrete algorithms. Many of the known randomized online algorithms are naturally divided into *phases* where each phase is processed and analyzed separately. Two examples are the algorithm for metrical task systems of Borodin et al. [4] and the marking algorithm for paging of Fiat et al. [5]. Since the phases are independent, we can obtain a high probability result (i.e., with a probability converging to 1 with an increasing number of phases; see Komm [9] for details on the marking algorithm). The definition of these phases, however, is specific to each problem and algorithm. Conversely, some other algorithms such as, e.g., the paging algorithm from Achlioptas et al. [2] and many workfunction-based algorithms use constructions that do not impose a division into phases.

First, we show that it is in general not meaningful to measure the probability of success with respect to the input size, which might be considered the straightforward approach; we demonstrate this for the paging problem, which implies the same statement for the *k*-server problem and task systems [3]. Consequently, we have to measure the success probability with respect to another parameter. We show that the profit or cost, depending on whether maximization or minimization problems are considered, of an optimal solution is a reasonable quantity for this purpose.

Our approach allows us to design randomized online algorithms that up to a factor of $(1+\varepsilon )$ have the expected performance with a probability tending to 1 with a growing size of the optimal profit or cost. We show that this technique is applicable for a wide range of online problems.

### Organization and Techniques

In Sect. 2, we define the class of symmetric online problems for which we will present the main result. Afterwards, in Sect. 2.1, we discuss why a straightforward approach towards defining “high probability” w.r.t. the input size must fail; this motivates the definition of high probability w.r.t. the profit (cost, respectively) of an optimal solution, given in Sect. 2.2. In Sect. 2.3, we provide a formal definition of the problem properties that we have identified to be crucial for designing online algorithms with high probability guarantees. This then enables us to provide a precise statement of the two main theorems which state that, for every problem that fulfills certain natural conditions, it is possible to transform an algorithm ${\textsc {Rand}}$ with constant expected competitive ratio *r* to an algorithm ${\textsc {Rand}}'$ having a competitive ratio of $(1+\varepsilon )r$ with high probability – with respect to the profit (cost) of an optimal solution. It should be noted that ${\textsc {Rand}}'$ has to compute optimal solutions to known prefixes of the given input, which may in principle result in a large, possibly even exponential, time complexity. However, for most online problems, such as paging and *k*-server, there exist efficient offline algorithms that compute optimal solutions.

Sections 3 and 4 are devoted to proving the main theorems for maximization and minimization problems, respectively. We partition the run of the algorithm into phases such that the loss incurred by the phase changes can be amortized. For minimization problems, in order to control the variance within one phase, however, we need to further subdivide the phases. By modeling the profit (cost) of single phases as dependent random variables, we obtain a submartingale (supermartingale), which enables us to apply the Azuma–Hoeffding inequality and thus to obtain the result.

After these investigations, we provide applications of the theorems in Sect. 5. In particular, we show that our result is applicable for task systems in Sect. 5.2. Task systems offer a general view of online problems including classical problems such as paging [2, 5, 14], *k*-server [10, 13], and the list update problem [14].

### Related Work

Classically, results concerning randomized online algorithms analyze their expected behavior; there are, however, a few exceptions. For example, Leonardi et al. [11] analyze the tail distribution of algorithms for call control problems, and Maggs et al. [12] deal with online distributed data management strategies that minimize the congestion in certain network topologies.

## Preliminaries

In this section, we fix the notation for online algorithms which we use throughout the paper. Before we start, we need to briefly discuss the way in which online problems and instances are formally defined. For our investigations, we have to be very careful about these definitions. In particular, in the literature one often refers to “an online problem” when actually a class of online problems is meant, which is parameterized by some problem-specific parameters. Let us give two examples of problems that we study later in the paper. When speaking about the paging problem, we really mean the class of paging problems determined by the cache size *k*; note that there is some inconsistency in the literature as this problem is usually referred to as “paging” (and not “*k*-paging”) while we speak of the “*k*-server problem.” For the latter, *k* denotes the number of servers that are moved in a metric space. However, *k* alone is not sufficient to specify a member from the class “*k*-server problems.” Additionally, we also need to specify the metric space (*M*, *d*), where *M* is a set of points and *d* is a metric distance function.

To completely define the above problems, we need to give even more information by speaking about how problem instances are initialized according to the parameters. For example, we need to specify how the cache is initialized for the paging problem or where the servers are located at the beginning when dealing with the *k*-server problem. We call this initialization the *initial situation*; for paging, the initial situation is a *k*-tuple $(s_1,\dots ,s_k)$ of distinct integers, which formalizes which pages are in the cache at the beginning. Formally, we thus have to speak of an instance of the $(k,(s_1,\dots ,s_k))$-paging problem. In general, such a parameterized online problem is given by $(\mathcal {C},\mathcal {I})$-$\Pi$ where $\mathcal {C}$ is a sequence of problem-specific parameters, $\mathcal {I}$ is a set of valid initial situations, and $\Pi$ is the name of the union of all of these problems. Formally, $\mathcal {I}$ is a set of valid assignments *I* to some of the parameters in $\mathcal {C}$, and the competitiveness guarantees of every algorithm for $\Pi$ must be satisfied for every $I\in \mathcal {I}$. Sometimes *I* is also considered to be part of the instance. To end this discussion, note that in the literature, the initial situation is at times called the initial configuration. In this paper, we choose another name to distinguish it from the configuration of an algorithm (Turing machine). In the following, we use the notation from the literature and omit $\mathcal {C}$. Nevertheless, the initial situation *I* plays an important role for us and it is given together with the actual input sequence *x*. Let us emphasize that if we say that some algorithm has some specific performance for a problem $\Pi$, this means that this performance must be guaranteed for *all* feasible choices of $\mathcal {C}$, *I*, and *x*.

We are now ready to define online algorithms on initial situations and input sequences. An *online algorithm* ${\textsc {Alg}}$ for $\Pi$ computes the output sequence ${\textsc {Alg}} (I,x)=y=(y_1,\dots ,y_n)$, where *I* is an initial situation, $x=(x_1,\dots ,x_n)$ is an input sequence, and $y_i$ is computed from $I,x_1,\dots ,x_i$. If $\Pi$ is a *maximization problem*, we denote the *profit* of the solution ${\textsc {Alg}} (I,x)$ by $profit (I,x,y) =profit (I,x,{\textsc {Alg}} (I,x))$; conversely, if $\Pi$ is a *minimization problem*, we denote the *cost* of ${\textsc {Alg}}$ ’s solution by $cost (I,x,y) =cost (I,x,{\textsc {Alg}} (I,x))$. For the ease of presentation, we refer to the tuple that consists of the initial situation and the input sequence, i.e., (*I*, *x*), as the *input of the problem*; also, we abbreviate $profit (I,x,{\textsc {Alg}} (I,x))$ by $profit ({\textsc {Alg}} (I,x))$ and $cost (I,x,{\textsc {Alg}} (I,x))$ by $cost ({\textsc {Alg}} (I,x))$, respectively. As already mentioned, the notion of an initial situation plays an important role in the relationship between different variants of the competitive ratio; although it is usually omitted, our definition imposes no restriction on the studied problems and algorithms.

A *randomized online algorithm* ${\textsc {Rand}}$ computes the output sequence ${\textsc {Rand}} ^{\phi }(I,x)=y=(y_1,\dots ,y_n)$ such that $y_i$ is computed from $\phi ,I,x_1,\dots ,x_i$, where $\phi$ is the content of a binary random string. By $profit ({\textsc {Rand}} (I,x))$ ($cost ({\textsc {Rand}} (I,x))$) we denote the random variable (over the probability space defined by $\phi$) expressing the profit (cost) of the solution ${\textsc {Rand}} ^{\phi }(I,x)$. When dealing with randomized online algorithms, we compare the expected outcome to the profit (cost) of an optimal offline solution. Throughout this paper, we assume an *oblivious adversary* and say that a randomized algorithm is *r*-competitive if there is a constant $\alpha$ such that, for every initial situation *I* and input sequence *x*,

$$\begin{aligned} \mathbb {E} \left[ profit ({\textsc {Rand}} (I,x)) \right] \ge profit ({\textsc {Opt}} (I,x))/r-\alpha \end{aligned}$$

if $\Pi$ is a maximization problem, and

$$\begin{aligned} \mathbb {E} \left[ cost ({\textsc {Rand}} (I,x)) \right] \le r\cdot cost ({\textsc {Opt}} (I,x)) +\alpha \end{aligned}$$

if $\Pi$ is a minimization problem. For formal reasons, we define the competitive ratio of any (randomized) online algorithm to be 1 if both *x* and *y* are empty.

### Defining High Probability w.r.t. the Input Size

In the sequel, we analyze the notion of *competitive ratio with high probability*. A natural way to define an event to have high probability would be to require that its probability to appear tends to 1 with increasing input length (i.e., the number of requests). However, in general this does not seem to be applicable. To illustrate this fact, we again take the well-known paging problem with cache size *k*, noting that the following hardness result immediately carries over to the *k*-server problem and task systems. Let us only consider the case of strict competitiveness, i.e., when the additive constant $\alpha$ is 0; the following remarks can also be found in Komm [9], where they are generalized to $\alpha \ge 0$. In what follows, we restrict ourselves to demand paging algorithms, i.e., online algorithms for paging that only evict a page from their respective cache if a page fault occurs. It is well known that, for every paging algorithm, there is a demand paging algorithm that is at least as good [3]. For every input sequence $x'$ of length *n* and every competitive ratio *r* and every *d*, there is an input sequence *x* of length *dn* formed by repeating every request *d* times. Consequently, for every online algorithm with some performance on *x*, we can design an online algorithm with the same performance on $x'$.

We now show that there is no randomized online algorithm for paging that achieves a competitive ratio of less than *k* with a probability approaching 1 with increasing *n*. Let $r<k$ and suppose that there is some $n_0\in \mathbb {N}$ and a randomized online algorithm ${\textsc {Rand}}$ that, for every input sequence *x* with $|x|=n\ge n_0$, is *r*-competitive with probability $1-1/f(n)$, for some function *f* that tends to infinity with growing *n*. By the above reasoning, there also is a randomized online algorithm that is *r*-competitive on every input sequence $x'$ independent of its length with the same probability. In particular, if ${\textsc {Rand}}$ exists, then there exists a randomized online algorithm ${\textsc {Rand}}'$ that is *r*-competitive on input sequences of length *k* with probability $1-1/f(n)$, for every *n*. Let there be $k+1$ pages (we simply denote them by $1,\dots ,k+1$) in total, and let the cache be initialized with pages $1,\dots ,k$. Now consider the following input sequence of length *k*. An adversary requests page $k+1$ at the beginning and some unique pages in the next $k-1$ time steps such that the same page is never requested in two consecutive time steps.

Clearly, there is an optimal solution with cost 1 that only causes a page fault with the first request. As for ${\textsc {Rand}}'$, in every time step in which a page fault occurs, it randomly chooses a page to evict to make space in the cache. Since the adversary knows the probability distribution ${\textsc {Rand}}'$ uses, without loss of generality, we assume that ${\textsc {Rand}}'$ chooses every page with the same probability. Note that there is a sequence $p_1,\dots ,p_k$ of “bad” choices that causes ${\textsc {Rand}}'$ to have cost *k*. In the first time step, ${\textsc {Rand}}'$ chooses the bad page with probability at least 1/*k*; with probability at least $1/k^2$, it chooses the bad pages in the first and the second time step and so on. Clearly, the probability that it chooses the bad sequence is at least $1/k^k$. However, this immediately contradicts that ${\textsc {Rand}}'$ has a strict competitive ratio better than the trivial one of *k* with probability $1+1/f(n)$ for arbitrarily large *n*.

### Defining High Probability w.r.t. the Optimal Solution

For the practical use of paging algorithms, the interesting input sequences are those where also the optimal algorithm causes more than constantly many page faults; otherwise, marking algorithms such as LRU and conservative algorithms such as FIFO also only cause a constant number of page faults [3, 14], and are therefore 1-competitive on these input sequences. Hence, it seems reasonable to define the term *high probability* with respect to the profit (cost) of an optimal solution. In what follows, we do this separately for maximization and minimization problems.

#### Definition 1

*(Competitive Ratio w.h.p. for Maximization Problems)* A randomized online algorithm ${\textsc {Rand}}$ is *r*-competitive *with high probability* (w.h.p. for short) if there is a constant $\alpha$ such that

$$\begin{aligned} \textstyle {\lim _{{\textsc {Opt}} (I,x)\rightarrow \infty }} Pr \left[ profit ({\textsc {Rand}} (I,x)) \le profit ({\textsc {Opt}} (I,x))/r-\alpha \right] = 0\;. \end{aligned}$$

As a consequence of our techniques, for our positive results, we are able to use a strong notion of high probability, requiring that, for every $\beta \ge 1$, the error probability is subpolynomial, i.e.,

1 $$\begin{aligned} Pr \left[ profit ({\textsc {Rand}} (I,x)) \le (profit ({\textsc {Opt}} (I,x))/r-\alpha \right] \le \left( 2+profit ({\textsc {Opt}} (I,x)) \right) ^{-\beta }\;.\nonumber \\ \end{aligned}$$

#### Definition 2

*(Competitive Ratio w.h.p. for Minimization Problems)* A randomized online algorithm ${\textsc {Rand}}$ is *r*-competitive *with high probability* (w.h.p. for short) if there is a constant $\alpha$ such that

$$\begin{aligned} \textstyle {\lim _{{\textsc {Opt}} (I,x)\rightarrow \infty }} Pr \left[ cost ({\textsc {Rand}} (I,x)) \ge r\cdot cost ({\textsc {Opt}} (I,x)) +\alpha \right] = 0\;. \end{aligned}$$

Also for our result on minimization problems, we can show that, for every $\beta \ge 1$, the error probability is subpolynomial, i.e.,

2 $$\begin{aligned} Pr \left[ cost ({\textsc {Rand}} (I,x)) \ge r\cdot cost ({\textsc {Opt}} (I,x)) +\alpha \right] \le \left( 2+cost ({\textsc {Opt}} (I,x)) \right) ^{-\beta }\;. \end{aligned}$$

Observe that for $cost ({\textsc {Opt}} (I,x)) \rightarrow \infty$, this value tends to 0.

Note that the purpose of the constant 2 in (1) and (2) is to properly handle inputs with a small (possibly zero) optimum.

### Symmetric Problems and High Probability

We start with formalizing a natural property of the profit (cost) associated with a given online problem.

#### Definition 3

*(Partition Function)* A *partition function* of an online problem is a non-negative function $\mathcal {P}$ such that, for every initial situation *I*, input sequence $x_1,\dots ,x_n$, and corresponding output sequence $y_1,\dots ,y_n$, we have

$$\begin{aligned} \text {measure}(I,(x_1,\dots ,x_n),(y_1,\dots ,y_n))=\sum _{i=1}^n\mathcal {P}(I, x_1,\dots ,x_i;y_1,\dots ,y_i)\;, \end{aligned}$$

for $$\text {measure}\in \{profit ,cost \}$$.

In other words, for a problem with a partition function, the profit (cost) of a solution is the sum of the profits (costs) of particular answers, and the profit (cost) of each answer is independent of the future input and output. For a fixed sequence of inputs and outputs, the partition function allows us to speak of the profit (cost) of a subsequence of the outputs. Note that any online problem for which input sequences may stop after each request has either a unique partition function or none, because the overall profit (cost) is fixed after each answer. Also note that a partition function exists unless it is possible to decrease the profit (cost) by appending more requests to the input sequence, which is not the case for most of the natural online problems.

In what follows, we further restrict the behavior, and it will be convenient to think in terms of the “profit or cost of a particular answer.” We may think of online problems that have a partition function as a separate class of problems. However, all further properties depend on specific partition functions and thus requiring a “partitionability” property would be redundant.

Note that for any problem with a partition function there is a natural notion of a state; for instance, it is the content of the cache for the paging problem, the position of the servers for the *k*-server problem, etc. Now we provide a general definition of this notion. By $a\cdot b$, we denote the concatenation of two sequences *a* and *b*; $\lambda$ denotes the empty sequence. An input $(I,x=(x_1,\dots ,x_n))$ is *feasible* with a solution $y=(y_1,\dots ,y_n)$ if starting from *I*, *x* is a request sequence that is in accord with the problem definition and for each *i*, $y_i$ is a feasible answer to the request $x_i$ with respect to *I*, $(x_1,\dots ,x_{i-1})$, and $(y_1,\dots ,y_{i-1})$.

#### Definition 4

*(State)* Consider a partition function $\mathcal {P}$, two initial situations *I* and $I'$, two input sequences $x=(x_1,\dots ,x_n)$ and $x'=(x_1',\dots ,x_m')$, and two output sequences $y=(y_1,\dots ,y_n)$ and $y'=(y'_1,\dots ,y'_m)$. The triples (*I*, *x*, *y*) and $(I',x',y')$ are equivalent if, for every input sequence $x''=(x''_1,\dots ,x''_p)$ and an output sequence $y''=(y_1'',\dots ,y_p'')$, the input $(I,x\cdot x'')$ is feasible with a solution $y\cdot y''$ if and only if the input $(I',x'\cdot x'')$ is feasible with a solution $y'\cdot y''$, and the profit (cost) of $y''$ according to $\mathcal {P}$ is the same for both solutions. A *state* *s* of the problem is an equivalence class over the triples (*I*, *x*, *y*).

For the paging problem, a state indeed formalizes the notion of a “fixed cache content.” To see this, consider two different cache contents. There is an input sequence that starts with requesting a page that is only contained in one of the two caches, and therefore there is a solution not evicting any page – which is only feasible for one of the two contents. Consequently, two different cache contents cannot be in the same state.

Consider some online problem $\Pi$, and let (*I*, *x*, *y*) be some triple in a state *s*. By ${\textsc {Opt}} _s(x')$ we denote an output sequence $y'$ such that $y \cdot y'$ is a feasible solution for the input $(I,x\cdot x')$ and, for every feasible solution $y \cdot y''$,

$$\begin{aligned} profit (I,x\cdot x',y\cdot y') \ge profit (I,x\cdot x',y\cdot y'') \end{aligned}$$

if $\Pi$ is a maximization problem, and

$$\begin{aligned} cost (I,x\cdot x',y\cdot y') \le cost (I,x\cdot x',y\cdot y'') \end{aligned}$$

if $\Pi$ is a minimization problem.

Note that due to the definition of states, the definition of ${\textsc {Opt}} _s(x')$ is independent of the chosen triple (*I*, *x*, *y*). We sometimes simplify notation and write $profit ({\textsc {Opt}} _s(x'))$ instead of $profit (I,x \cdot x',y \cdot {\textsc {Opt}} _s(x')) - profit (I,x,y)$ or $cost ({\textsc {Opt}} _s(x'))$ instead of $cost (I,x \cdot x',y \cdot {\textsc {Opt}} _s(x')) - cost (I,x,y)$, respectively, as it is sufficient to know *s* and $x'$ in order to determine the value of the function.

#### Definition 5

*(Initial State)* A state *s* is called an *initial state* if and only if it contains some triple $(I,\lambda ,\lambda )$.

We chose this definition of states as it covers best the properties of online computation as we need them in our two main theorems. Once again using paging as an example, we require that every cache content is feasible at the beginning; for *k*-server we allow the servers to be placed anywhere in the metric space before the first request. An alternative definition could use task systems with infinitely many states, but the description would become less intuitive; we will return to task systems in Sect. 5.2.

Intuitively, a state from Definition 4 encapsulates all information about the ongoing computation of the algorithm that is relevant for evaluating the efficiency of the future processing. Usually, the state is naturally described in the problem-specific domain (e.g., the aforementioned current cache content or server positions). Similar to our discussion on initial situations, we want to emphasize that a state is independent of the concrete algorithm. The internal state of an algorithm is a different notion since it may, e.g., behave differently if the starting request had some particular value. The following properties are crucial for our approach towards probability amplification.

#### Definition 6

*(Opt-Boundedness)* An online problem is called ${\textsc {Opt}}$ *-bounded* if there is a constant *B* and a partition function such that

$$\begin{aligned} \forall s,s',x:|\text {measure}({\textsc {Opt}} _s(x))-\text {measure}({\textsc {Opt}} _{s'}(x))|\le B\;, \end{aligned}$$

for $$\text {measure}\in \{profit ,cost \}$$.

In simple terms, *B* bounds the different profits (costs) between two optimal solutions for different states on a fixed input. Again using paging as an example, we can safely set $B=k$ since starting with two different caches can make at most a difference of *k* page faults.

#### Definition 7

*(Symmetric Problem)* An online problem is called *symmetric* if it has a partition function for which every state is initial.

Note that for symmetric problems, it follows that every sequence of requests is a feasible input sequence; this includes single requests. In particular, the input may end after each time step. Once more consider paging, for which every sequence of pages is a feasible input sequence, or *k*-server, where every sequence of points in the given metric space is feasible. Formally, every problem with a partition function may be transformed into a symmetric one simply by redefining the set of initial states. However, this transformation may significantly change the properties of the problem.

In the following two sections, we will prove the two main theorems of this paper, namely that, under certain conditions, the expected competitive ratio of symmetric problems can be achieved w.h.p. according to Definitions 1 and 2 (actually, even (1) and (2)), respectively. Section 3 studies the simpler case of maximization problems, whereas for minimization problems, investigated in Sect. 4, we need to make an additional assumption.

## The Main Theorem for Maximization Problems

In this section, we study online maximization problems according to Definitions 6 and 7, and prove the first half of our main result. The counterpart will be proven in the subsequent section.

### Theorem 1

Consider an online maximization problem $\Pi$ that is ${\textsc {Opt}}$-bounded and symmetric according to a common partition function. Suppose there is a randomized online algorithm ${\textsc {Rand}}$ for $\Pi$ with constant expected competitive ratio *r*. Then, for each constant $\varepsilon >0$, there is a randomized online algorithm ${\textsc {Rand}}'$ with competitive ratio $(1+\varepsilon )r$ w.h.p.

At first, we provide a high-level informal description of the ideas behind our proof. The algorithm ${\textsc {Rand}}'$ simulates ${\textsc {Rand}}$ and, on some specific places, performs a *reset* operation: if a part $x'$ of the input sequence has been processed so far, and a corresponding output sequence $y'$ has been produced, $(I,x',y')$ belongs to the same state as $(I',\lambda ,\lambda )$, for some initial situation $I'$ due to the symmetry of $\Pi$; hence, ${\textsc {Rand}}$ can be restarted by ${\textsc {Rand}}'$ from $I'$.

The general idea to boost the probability of acquiring a high profit is to perform a reset sufficiently often. Therefore, the input is partitioned into *phases* in such a way that ${\textsc {Rand}}'$ can deterministically decide the boundaries of the phases. ${\textsc {Rand}}'$ performs a reset at the end of each phase. Then again, a reset may cause a bounded loss of expected profit; we consequently have to ensure that the phases are long enough, i.e., their optimum profit is large enough, so as to amortize this overhead.

Since we design the phase boundaries in such a way that ${\textsc {Rand}}'$ is aware when it processes the last request of a phase, we can make ${\textsc {Rand}}'$ process the last request greedily, i.e., maximize the profit without paying attention to the resulting state. As we can reset the simulated algorithm ${\textsc {Rand}}$ in any state, this will not interfere with the subsequent run of ${\textsc {Rand}}$. We will need this property later to have a deterministic upper bound on the difference between the optimal profit and the profit of ${\textsc {Rand}}'$ on a single phase.

${\textsc {Rand}}'$ will make sure that the number of phases grows sufficiently quickly with growing profit of the optimal solution. We do not, however, assume any upper bound on the profit of a single request, which means that such a condition cannot be guaranteed. To work around this problem, we first define *heavy requests* as requests that can be, in some state, answered such that the gained profit is larger than a certain threshold *T*. The ${\textsc {Opt}}$-boundedness then guarantees that a heavy request can always be answered with profit at least $T-B$ (where *B* denotes the constant from Definition 6). Thus, if *T* is sufficiently large, the greedy answer is almost optimal—the ratio between the greedy answer and the answer of the optimal solution is arbitrarily close to 1. If we make sure that the last request of a phase is always a heavy request, then ${\textsc {Rand}}'$ answers all such requests greedily.

Afterwards, we consider two cases. On the one hand, if a very large fraction of the profit of the optimal solution stems from heavy requests, it does not matter what the algorithm does on the non-heavy requests, because answering all heavy requests greedily is sufficient to ensure competitive ratio *r*. On the other hand, if at least some small but fixed fraction of the optimal solution stems from non-heavy requests, we can guarantee that the number of phases grows linearly in the profit of the optimal solution.

We proceed to formalize these ideas. From now on let us consider $\varepsilon$, *r*, $\alpha$, and *B* to be fixed constants. Recall that ${\textsc {Rand}}$ is *r*-competitive in expectation with an additive constant $\alpha$. Our goal is to design the randomized online algorithm ${\textsc {Rand}}'$ that is $(1+\varepsilon )r$-competitive with high probability and additive constant $\alpha '$. ${\textsc {Rand}}'$ is parameterized by parameters *T* and *C*, which depend on $\varepsilon$, *r*, $\alpha$, and *B*. As described above, the parameter *T* controls the threshold for heavy requests and *C* controls the length of the phases, such that the optimal profit of one phase is roughly *C*. For the remainder of our proof, we can fix *C* to any value that satisfies

3 $$\begin{aligned} C > B\cdot \left( 1 + (r+1)\cdot \frac{1+\varepsilon }{\varepsilon }\right) + \alpha \cdot r\cdot \frac{1+\varepsilon }{\varepsilon }\;, \end{aligned}$$

and for *T* we require both

4 $$\begin{aligned} T > C + B \end{aligned}$$

and

5 $$\begin{aligned} T > B\cdot \left( 2 + \frac{r}{r-1}\right) \;. \end{aligned}$$

The additive constant $\alpha '$ of ${\textsc {Rand}}'$ depends on $\varepsilon$, *r*, $\alpha$, *B*, *C*, and *T* only; it will be fixed later in the proof.

### Definition 8

Let $x_i$ be a request. We say that $x_i$ is a *heavy request* if there is some initial situation $I'$ and some answer $y_i$ such that $profit (I',(x_i),(y_i)) \ge T$; otherwise, we say that $x_i$ is a *light request*.

Consider an initial situation *I* and an input sequence $x=(x_1,\dots ,x_n)$. Let $O_i$ be the optimal profit of the input sequence truncated to requests before $x_i$, i.e., the optimal profit of $(I,(x_1,\ldots ,x_{i - 1}))$; the optimal profit of the empty sequence is defined to be zero, i.e., $O_1:=0$. Since the profit function is non-negative, we have $O_i\le O_{i+1}$.

We define the boundaries of the phases such that phase *i* starts at request $$x_{n_i}$$, where $$n_1:=1$$, and $$n_{i+1}\le n+1$$ is the smallest number such that $$O_{n_{i+1}} \ge O_{n_i} + C$$. If no such value exists for some *i*, then $$i-1$$ is the last phase, and the suffix of the input $$x_{n_i},\ldots , x_n$$ is not part of any phase. Note that the phase boundaries can be computed by the online algorithm itself in a deterministic way, i.e., $${\textsc {Rand}}'$$ can determine when it is processing the last request of the phase. Now $${\textsc {Rand}}'$$ simulates $${\textsc {Rand}}$$ on every request that is not the last one in the current phase. For the last request of the phase, $${\textsc {Rand}}'$$ answers greedily: if the current state is equivalent to some $$(I',\lambda ,\lambda )$$ and the processed request is $$x_j$$, it outputs $$y_j$$ such that $$profit (I',(x_j),(y_j)) $$ is as large as possible. Afterwards $${\textsc {Rand}}'$$ restarts $${\textsc {Rand}}$$. Note that $${\textsc {Rand}}'$$ is not able to detect when it is processing requests past the last phase; however, we do not care about the behavior of $${\textsc {Rand}}'$$ on this part as long as it produces some valid output.

Observe that (4) guarantees that every heavy request is the last request of some phase: as any heavy request $x_i$ can be answered with a profit of at least $T-B$ in every state, $O_i \ge O_{i-1} + T-B > O_{i-1} + C$.

Now consider any fixed optimal solution ${\textsc {Opt}} (I, x)$, and let $profit _{\text {h}}({\textsc {Opt}} (I,x))$ be the profit of ${\textsc {Opt}} (I, x)$ created by heavy requests only. Let $\rho :=1/r + B/(T-2B)$. It follows from (5) that $\rho < 1$. We distinguish two cases.

*Case 1.* Assume that $profit _{\text {h}}({\textsc {Opt}} (I,x)) \ge \rho \cdot profit ({\textsc {Opt}} (I,x))$, i.e., a large fraction of the optimal profit stems from heavy requests. Due to the ${\textsc {Opt}}$-boundedness property, we may assume that each heavy request is answered in ${\textsc {Opt}} (I,x)$ with a profit of at least $T-2B$: as already noted, in any state it is possible to answer a heavy request with a profit of at least $T-B$, and the optimal solution cannot lose more than *B* compared to that (or there would be a better solution, which is a direct contradiction). Thus, there are at most $h:=profit ({\textsc {Opt}} (I,x)) / (T-2B)$ heavy requests. Since ${\textsc {Rand}}'$ answers all heavy requests greedily, it may lose at most *B* profit on each heavy request compared to the optimal solution. Therefore, the profit of ${\textsc {Rand}}'$ on (*I*, *x*) is at least

$$\begin{aligned} profit _{\text {h}}({\textsc {Opt}} (I,x))-hB\ge profit ({\textsc {Opt}} (I,x)) \left( \rho -\frac{B}{T-2B} \right) = profit ({\textsc {Opt}} (I,x)) / r\;, \end{aligned}$$

which means that ${\textsc {Rand}}'$ is *r*-competitive with additive constant 0, i.e., for any $\alpha ' \ge 0$, with probability 1.

*Case 2.* Assume that $profit _{\text {h}}({\textsc {Opt}} (I,x)) < \rho \cdot profit ({\textsc {Opt}} (I,x))$, i.e., the profit of more than $(1-\rho )\cdot profit ({\textsc {Opt}} (I,x))$ stems from light requests. Then let *U*(*i*, *j*) be the profit of ${\textsc {Opt}} (I,x)$ gained on the requests $x_i,\ldots ,x_j$. This partial profit is closely related to the prefix-optimum function $O_i$.

### Observation 1

We have $U(i,j) \le O_{j+1} - O_i + B$ and $U(i,j) \ge O_{j+1} - O_i - B$.

### Proof

On any given prefix, ${\textsc {Opt}}$ cannot be better than the optimal solution on that prefix, i.e., $U(1,j) \le O_{j+1}$. Moreover, ${\textsc {Opt}}$ cannot be much worse than the prefix-optimal solution due to ${\textsc {Opt}}$-boundedness, i.e., $U(1,j) \ge O_{j+1} -B$. Due to $U(i,j) = U(1,j) - U(1,i-1)$, the claim follows. $\square$

In what follows, let *k* denote the number of phases. We proceed to bound the profit of requests past the last phase. Due to $O_{n+1}-O_{k+1} < C$, we have that

6 $$\begin{aligned} U(n_{k+1},n)<C+B\;. \end{aligned}$$

Next we provide bounds on *k*. In a single phase, there cannot be too much profit gained by light requests by ${\textsc {Opt}}$. Indeed, by definition, $O_{n_{i+1}-1} - O_{n_i} < C$; thus, due to Observation 1, $U(n_i, n_{i+1}-2) < C +B$. The profit gained by ${\textsc {Opt}}$ by the light requests in phase *i* is either $U(n_i, n_{i+1}-2)$ if the last request of phase *i* is heavy, or $U(n_i, n_{i+1} -1)$ if the last request is light. In the latter case, however, the profit gained by the last request is at most *T*. Consequently, the profit gained by light requests by ${\textsc {Opt}}$ in any phase is less than $C+B+T$. The same argument holds for requests past the last phase due to (6). Since we have a lower bound on the total profit gained by light requests in ${\textsc {Opt}} (I,x)$, we have that

7 $$\begin{aligned} k > profit ({\textsc {Opt}} (I,x)) \frac{1-\rho }{C+B+T} - 1\;. \end{aligned}$$

As a result, for fixed parameters *r*, *C*, *B*, and *T*, the number of phases grows linearly with growing profit of the optimal solution of (*I*, *x*).

To provide an upper bound on the number of phases, note that there is a lower bound on the profit gained by ${\textsc {Opt}}$ within a single phase. As $O_{n_{i+1}} - O_{n_i} \ge C$, we have that $U(n_i, n_{i+1}-1)\ge C-B$. Thus, there are

8 $$\begin{aligned} k\le U(1, n_{k+1}-1)/(C-B) \end{aligned}$$

phases.

Now we are going to discuss the profit of ${\textsc {Rand}}'$ on the input (*I*, *x*). Let $S_i$ be a random variable denoting the state of the problem (according to Definition 4) just before processing request $x_i$, and let *W*(*i*, *j*) with $i\le j$ be a random variable denoting the profit of ${\textsc {Rand}}'$ incurred on the request sequence $x_i,\dots ,x_j$; let $W(i, i-1):=0$.

Since ${\textsc {Rand}}'$ answers the last request of each phase *i* greedily, it gains a profit of at least $U(n_{i+1}-1, n_{i+1}-1)-B$.

### Observation 2

For each $i\in \{1,\ldots ,k\}$, we have $W(n_{i+1}-1, n_{i+1}-1) \ge U(n_{i+1}-1, n_{i+1}-1)-B$.

The following claim follows directly from the definitions.

### Claim 1

If ${\textsc {Rand}}'$ performs a reset just before processing $x_i$, then $S_i$ captures all the information from the past that *W*(*i*, *j*) depends on. In particular, for any state *s* and any $l_1\le l_2 < i$, the random variables $W(i,j)\mid S_i=s$ and $W(l_1,l_2)\mid S_i=s$ are independent.

In the following, we simplify our notation by using $U_i:=U(n_i, n_{i+1}-1)$ to denote the (deterministic) profit of ${\textsc {Opt}} (I,x)$ in phase *i* and $W_i:=W(n_i, n_{i+1}-1)$ to denote the profit of ${\textsc {Rand}}' (I,x)$ in phase *i*; note that $W_i$ is a random variable.

We consider random variables $Z_0,\dots ,Z_k$ such that $Z_i$ expresses the total expected profit of ${\textsc {Rand}}'$ if the outcomes of phases up to *i* are already fixed. Our goal is to show that they form a submartingale and apply the Azuma–Hoeffding inequality to bound the probability that ${\textsc {Rand}}'$ is much worse than the expectation. However, the Azuma–Hoeffding theorem requires a bound on $Z_{i+1}-Z_i$ with probability 1, which is not straightforward to get if there are requests of arbitrarily large profit. Therefore, we need to consider the heavy requests separately. In particular, we define the expected profit of a phase $i\in \{1,\ldots ,k\}$ as an *r*-approximation of all requests except the last one while allowing only a constant loss on the last request. We set

$$\begin{aligned} \mu _i :=U(n_i, n_{i+1} - 2)/r + U(n_{i+1}-1, n_{i+1}-1) - \alpha -B(1+1/r)\;, \end{aligned}$$

and define the random variables as

$$\begin{aligned} Z_i :=\sum _{j=1}^i W_j + \sum _{j=i+1}^k \mu _j\;. \end{aligned}$$

First, we show that $\mu _i$ is a lower bound on the expected profit of ${\textsc {Rand}}'$ in phase *i*.

### Lemma 1

For any *i* and *s*, we have $\mathbb {E} [W_i \mid S_{n_i}=s]\ge \mu _i$.

### Proof

We consider the last request of the phase separately. Recall that $u_i:=U(n_i,n_{i+1}-2)$ denotes the profit gained by ${\textsc {Opt}} (I,x)$ in phase *i* on all requests except the last one. By ${\textsc {Opt}}$-boundedness, regardless of the starting state *s*, it is possible to gain a profit of $u_i-B$ on these requests. Hence, the simulated algorithm ${\textsc {Rand}}$ gains an expected profit of at least $(u_i-B)/r - \alpha$. Since ${\textsc {Rand}}'$ performed a reset at the start of phase *i*, i.e., just before processing request $n_i$ and then simulated ${\textsc {Rand}}$, this is also the expected profit of ${\textsc {Rand}}'$. By Observation 2, the last request of phase *i* is answered with a profit of at least $U(n_{i+1}-1, n_{i+1}-1) - B$. Summing these two expected profits yields the claim. $\square$

Second, we prove that $Z_0,\dots ,Z_k$ form a bounded submartingale, and then use the Azuma–Hoeffding inequality to conclude that $Z_k$ is unlikely to be much smaller than $Z_0$. Since $Z_k=W(1,n)$ is equal to the profit of ${\textsc {Rand}}'$ on the complete input sequence, this allows us to bound the probability that ${\textsc {Rand}}'$ is significantly worse than $\sum _{i=1}^k \mu _i$.

### Lemma 2

The sequence $Z_0,\dots ,Z_k$ is a submartingale.

### Proof

We have to show that, for each *i* with $0\le i\le k$, we have $\mathbb {E} [Z_{i+1}\mid Z_0,\dots ,Z_i]\ge Z_i$. From the definition of the $Z_i$’s it follows that $Z_{i+1}-Z_i=W_{i+1} -\mu _{i+1}$. Consider any elementary event $\xi$ from the probability space, and let $Z_i(\xi )=z_i$, for $i=0,\dots ,k$, be the values of the corresponding random variables. We have

$$\begin{aligned}&\mathbb {E} [Z_{i+1}\mid Z_0,\dots ,Z_i](\xi )\\&\quad = \mathbb {E} [ Z_{i+1}\mid Z_0=z_0,\dots ,Z_i=z_i]\\&\quad = \mathbb {E} [ Z_i+W_{i+1}-\mu _{i+1}\mid Z_0=z_0,\dots ,Z_i=z_i]\\&\quad = z_i-\mu _{i+1}+\mathbb {E} [W_{i+1}\mid Z_0=z_0,\dots ,Z_i=z_i]\\&\quad = z_i-\mu _{i+1}+\sum _s\mathbb {E} [W_{i+1}\mid Z_0=z_0,\dots ,Z_i=z_i,S_{n_{i+1}}\!=s]\\&\qquad \cdot Pr [S_{n_{i+1}}\!=s \mid Z_0=z_0,\dots ,Z_i=z_i]\\&\quad = z_i-\mu _{i+1}+\sum _s\mathbb {E} [W_{i+1}\mid S_{n_{i+1}}\!=s]\cdot Pr [S_{n_{i+1}}\!=s \mid Z_0=z_0,\dots ,Z_i=z_i]\\&\quad \ge z_i-\mu _{i+1}+ \mu _{i+1} \sum _sPr [S_{n_{i+1}}=s \mid Z_0=z_0,\dots ,Z_i=z_i]=z_i=Z_i(\xi )\;, \end{aligned}$$

where the last inequality is a consequence of Lemma 1. $\square$

Now we use the following special case of the Azuma–Hoeffding inequality [1, 6].

### Lemma 3

(Azuma, Hoeffding) Let $Z_0,Z_1,\dots , Z_k$ be a submartingale, such that $|Z_{i+1}-Z_i|<\gamma$. Then for any positive real *t*,

$$\begin{aligned} Pr [ Z_0 - Z_k \ge t]\le \exp \left( -\frac{t^2}{2k\gamma ^2}\right) \;. \end{aligned}$$

As mentioned above, in order to apply Lemma 3, we need to provide a bound on $|Z_{i+1}-Z_i|$ with probability 1.

### Claim 2

For any *i* with $1\le i\le k$, it holds that $|Z_i-Z_{i-1}|<\gamma$, where $\gamma :=C + B(4+1/r)$.

### Proof

By definition, $Z_i-Z_{i-1}=W_i - \mu _i = W_i - U(n_i, n_{i+1} - 2)/r - U(n_{i+1}-1, n_{i+1}-1) + \alpha + B(1+1/r)$. We provide the lower bound $-C-2B$ and the upper bound $B(3+1/r)$ on $Z_i-Z_{i-1}$ separately. Using that $r>1$, $\alpha \ge 0$, $B>0$, the fact that the profit function is non-negative, Observation 1, the definition of $O_i$, and Observation 2, we obtain

$$\begin{aligned}&Z_i-Z_{i-1}> W_i - U(n_i, n_{i+1} - 2) - U(n_{i+1}-1, n_{i+1}-1) \\&\quad = W(n_i, n_{i+1}-2) + W(n_{i+1}-1, n_{i+1}-1) - U(n_i, n_{i+1} - 2) - U(n_{i+1}-1, n_{i+1}-1) \\&\quad> - U(n_i, n_{i+1} - 2) + W(n_{i+1}-1, n_{i+1}-1) - U(n_{i+1}-1, n_{i+1}-1) \\&\quad> - (O_{n_{i+1} - 1} - O_{n_i} + B) + W(n_{i+1}-1, n_{i+1}-1) - U(n_{i+1}-1, n_{i+1}-1) \\&\quad> -C - B + W(n_{i+1}-1, n_{i+1}-1) - U(n_{i+1}-1, n_{i+1}-1)\\&\quad > -C - 2B \ge -\gamma \;. \end{aligned}$$

For the upper bound, we use the non-negativity of the profit function to get

$$\begin{aligned} Z_i-Z_{i-1}\le & {} W(n_i, n_{i+1} - 2) + W(n_{i+1}-1, n_{i+1}-1) - U(n_{i+1}-1, n_{i+1}-1) \\&+ \alpha + B(1+1/r)\;. \end{aligned}$$

Due to ${\textsc {Opt}}$-boundedness, ${\textsc {Opt}}$ can answer request $n_{i+1}-1$ with a profit of at least $W(n_{i+1}-1, n_{i+1}-1)(\xi )-B$ for any elementary event $\xi$. As ${\textsc {Opt}}$ is optimal on the complete input (*I*, *x*), it must answer this request with a profit of at least $W(n_{i+1}-1, n_{i+1}-1)(\xi )-2B$ due to ${\textsc {Opt}}$-boundedness. Hence, $W(n_{i+1}-1, n_{i+1}-1) - U(n_{i+1}-1, n_{i+1}-1) \le 2B$. Therefore,

$$\begin{aligned} Z_i-Z_{i-1} \le W(n_i, n_{i+1} - 2) + \alpha + B(3+1/r)\;. \end{aligned}$$

Furthermore, $W(n_i, n_{i+1} - 2) \le O_{n_{i+1}-1} - O_{n_i} + B < C+B$; otherwise, a solution with a profit of more than $O_{n_{i+1}-1}$ can be constructed on the input sequence $x_1,\dots , x_{n_{i+1}-2}$ due to ${\textsc {Opt}}$-boundedness, which is a contradiction to the definition of $O_{n_{i+1}-1}$. As a result, we obtain

$$\begin{aligned} Z_i-Z_{i-1} < C + \alpha + B(4+1/r) = \gamma \;, \end{aligned}$$

which proves the claim. $\square$

We are now ready to apply Lemma 3 in order to finally bound the probability that ${\textsc {Rand}}'$ does not produce a $(1+\varepsilon )r$-competitive result.

### Lemma 4

If $k\ge 1$, then there are $\nu _1 > 0$, $\nu _2$, and $\alpha ' \ge 0$ that depend on *r*, $\varepsilon$, *B*, *C*, and *T* only, such that

$$\begin{aligned} Pr \left[ profit ({\textsc {Rand}}' (I,x)) \le \frac{profit ({\textsc {Opt}} (I,x))}{r(1+\varepsilon )}-\alpha ' \right] \le \exp \left( -\nu _1\cdot profit ({\textsc {Opt}} (I,x)) +\nu _2 \right) \;. \end{aligned}$$

### Proof

Note that $profit ({\textsc {Rand}}' (I,x)) \ge \sum _{j=1}^k W_j = Z_k$. Therefore,

$$\begin{aligned}&Pr \left[ profit ({\textsc {Rand}}' (I,x)) \le \frac{profit ({\textsc {Opt}} (I,x))}{r(1+\varepsilon )}-\alpha ' \right] \\&\quad \le Pr \left[ Z_k\le \frac{profit ({\textsc {Opt}} (I,x))}{r(1+\varepsilon )}-\alpha ' \right] \\&\quad = Pr \left[ Z_0 - Z_k\ge Z_0 - \frac{profit ({\textsc {Opt}} (I,x))}{r(1+\varepsilon )}+\alpha ' \right] \;. \end{aligned}$$

Now let

$$\begin{aligned} \alpha ' :=\frac{C+B}{r(1+\varepsilon )} \end{aligned}$$

and

$$\begin{aligned} t:=Z_0 -\frac{profit ({\textsc {Opt}} (I,x))}{r(1+\varepsilon )}+\alpha ' = \sum _{i=1}^k \mu _i -\frac{profit ({\textsc {Opt}} (I,x))}{r(1+\varepsilon )}+\frac{C+B}{r(1+\varepsilon )}\;. \end{aligned}$$

Our goal is to apply Lemma 3; thus, we need to ensure that $t>0$. Since $r>1$, we have $\mu _i \ge U_i/r -\alpha -B(1+1/r)$. Using (6), we have that $profit ({\textsc {Opt}} (I,x)) < U(1, n_{k+1}-1) + C+B$. We get

$$\begin{aligned} t&= \sum _{i=1}^k \mu _i -\frac{profit ({\textsc {Opt}} (I,x))}{r(1+\varepsilon )}+\frac{C+B}{r(1+\varepsilon )} \\&\ge \frac{U(1, n_{k+1}-1)}{r} -k\left( \alpha +B\cdot \frac{r+1}{r}\right) -\frac{U(1, n_{k+1}-1) + C+B}{r(1+\varepsilon )}+\frac{C+B}{r(1+\varepsilon )} \\&= \frac{U(1, n_{k+1}-1)}{r} -k\left( \alpha +B\cdot \frac{r+1}{r}\right) -\frac{U(1, n_{k+1}-1)}{r(1+\varepsilon )} \\&= U(1, n_{k+1}-1)\cdot \frac{\varepsilon }{r(1+\varepsilon )} -k\left( \alpha +B\cdot \frac{r+1}{r}\right) \;. \end{aligned}$$

By (8), $k(C-B) \le U(1, n_{k+1}-1)$, and with this, we obtain

$$\begin{aligned} t&\ge k(C-B)\cdot \frac{\varepsilon }{r(1+\varepsilon )} -k\left( \alpha +B\cdot \frac{r+1}{r}\right) \\&= k\left( \frac{(C-B)\varepsilon }{r(1+\varepsilon )} - \left( \alpha +B\cdot \frac{r+1}{r}\right) \right) \\&= k\frac{(C-B)\varepsilon - \alpha \cdot r(1+\varepsilon ) - B(r+1)(1+\varepsilon )}{r(1+\varepsilon )}\\&= k\frac{C\cdot \varepsilon -B(\varepsilon + (r+1)(1+\varepsilon )) - \alpha \cdot r(1+\varepsilon )}{r(1+\varepsilon )}\\&= k\cdot \nu '\;, \end{aligned}$$

where

$$\begin{aligned} \nu ':=\frac{C\cdot \varepsilon -B(\varepsilon + (r+1)(1+\varepsilon )) - \alpha \cdot r(1+\varepsilon )}{r(1+\varepsilon )}\;. \end{aligned}$$

Hence, to ensure that $t>0$, it is sufficient to make sure that $\nu '>0$, i.e.,

$$\begin{aligned}&0&< C\cdot \varepsilon -B(\varepsilon + (r+1)(1+\varepsilon )) - \alpha \cdot r(1+\varepsilon ) \\ \iff \quad&C\cdot \varepsilon&> B(\varepsilon + (r+1)(1+\varepsilon )) + \alpha \cdot r(1+\varepsilon ) \\ \iff \quad&C&> B\left( 1 + (r+1)\frac{1+\varepsilon }{\varepsilon }\right) + \alpha \cdot r\frac{1+\varepsilon }{\varepsilon }\;, \end{aligned}$$

which is guaranteed by (3).

Applying Lemma 3, we get

$$\begin{aligned} Pr \left[ profit ({\textsc {Rand}}' (I,x)) \le \frac{profit ({\textsc {Opt}} (I,x))}{r(1+\varepsilon )}-\alpha ' \right] \le \exp \left( -\frac{t^2}{2k\gamma ^2}\right) \le \exp \left( -\frac{k \nu '}{2\gamma ^2}\right) \;. \end{aligned}$$

By (7), we have that

$$\begin{aligned}&Pr \left[ profit ({\textsc {Rand}}' (I,x)) \le \frac{profit ({\textsc {Opt}} (I,x))}{r(1+\varepsilon )}-\alpha ' \right] \\&\quad \le \exp \left( -\left( profit ({\textsc {Opt}} (I,x)) \frac{1-\rho }{C+B+T} -1\right) \cdot \frac{ \nu '}{2\gamma ^2}\right) \;. \end{aligned}$$

Finally, by setting

$$\begin{aligned} \nu _1:=\frac{1-\rho }{C+B+T}\cdot \frac{ \nu '}{2\gamma ^2} \end{aligned}$$

and

$$\begin{aligned} \nu _2:=\frac{\nu '}{2\gamma ^2} \end{aligned}$$

the claim of the lemma follows. $\square$

The main result then directly follows from Lemma 4.

### Proof

(of Theorem 1) If $profit ({\textsc {Opt}} (I,x))$ is sufficiently large, we have $k>1$ and can apply Lemma 4, yielding that, for any $\beta$, $\nu _1>0$, and $\nu _2$,

$$\begin{aligned} \exp \left( -\nu _1\cdot profit ({\textsc {Opt}} (I,x)) +\nu _2\right) \le \left( 2+cost ({\textsc {Opt}} (I,x)) \right) ^{-\beta }\;. \end{aligned}$$

The behavior of ${\textsc {Rand}}'$ on inputs where the optimal solution is smaller can be hidden in the additive constant $\alpha '$. $\square$

## The Main Theorem for Minimization Problems

In this section, we proof the second part of our main result. However, as opposed to maximization problems, in the case of minimization, we need to impose another yet very natural restriction on the problems studied.

### Definition 9

*(Request-Boundedness)* An online problem $\Pi$ is called *request-bounded* if, for some constant *F*, it has a partition function $\mathcal {P}$ such that

$$\begin{aligned} \forall I,x,y,i:\mathcal {P}(I,x_1,\dots ,x_i;y_1,\dots ,y_i)\le F\;. \end{aligned}$$

We say that $\Pi$ is *request-bounded* according to $\mathcal {P}$.

We conjecture that it is possible to drop this requirement using arguments somewhat similar to those used in Sect. 3. However, it seems to be far from trivial to formulate a straightforward adaption for the case that there is a large total cost in heavy requests. In any case, again taking paging and *k*-server on finite metric spaces as examples, request-boundedness is indeed satisfied.

Besides that, we can somewhat adapt the ideas used for maximization problems to achieve an analogous result for minimization problems. However, even with the request-boundedness property, there remains one issue that prevents us from applying them in a straightforward fashion. The reason is that, in minimization problems, there is no deterministic bound on the difference between the algorithm’s solution and the optimum for a single phase. While in the maximization setup, the worst case is that the algorithm gains nothing, while the optimum cost is fixed, in the minimization setup, the algorithm can, with small probability, incur some arbitrarily large cost. To avoid this problem, we introduce the concept of *subphases*. Whenever the simulated randomized algorithm incurs too large of a cost within a single phase, we introduce an extra reset. We are then able to bound the probability that another reset happens using Markov’s inequality, and then ensure that the probability of a large number of subphases decreases exponentially in the number of subphases. This allows us to split the probability of a bad result in the final analysis into two cases: either there are sufficiently many subphases, or we can apply the Azuma–Hoeffding inequality for a fixed bound on a phase cost increase.

### Theorem 2

Consider an online minimization problem $\Pi$ that is request-bounded, ${\textsc {Opt}}$-bounded, and symmetric according to a common partition function. Suppose there is a randomized online algorithm ${\textsc {Rand}}$ for $\Pi$ with constant expected competitive ratio *r*. Then, for each constant $\varepsilon >0$, there is a randomized online algorithm ${\textsc {Rand}}'$ with competitive ratio $(1+\varepsilon )r$ w.h.p..

As in Sect. 3, ${\textsc {Rand}}'$ simulates ${\textsc {Rand}}$ and performs *reset* operations at specific places. Again, the general idea is to boost the probability of (this time) acquiring a low cost by performing a reset each time the algorithm incurs too much cost. To this end, the request sequence is again partitioned into *phases* of a fixed optimal cost; this time, however, each phase may be further cut into *subphases* based on the cost incurred by ${\textsc {Rand}}'$ so far. A reset may cause an additional expected cost of $r \cdot B+\alpha$ for the subsequent phase compared to an optimal solution starting from another state, where *B* is the constant of the ${\textsc {Opt}}$-boundedness property (Definition 6). We therefore have to ensure that the phases are long enough so as to amortize this overhead.

From now on let us consider $\varepsilon$, *r*, *B*, *F*, and $\alpha$ to be fixed constants. Recall that *F* originates from the request-boundedness property of the online minimization problem at hand (Definition 9) and ${\textsc {Rand}}$ is *r*-competitive in expectation with an additive constant $\alpha$. The algorithm ${\textsc {Rand}}'$ is parameterized by two parameters *C* and *D* which depend on $\varepsilon$, *r*, *B*, *F*, and $\alpha$. These parameters control the lengths of the phases and subphases, respectively, such that $C+F$ delimits the optimal cost of one phase and $D+F$ delimits the cost of the solution computed by ${\textsc {Rand}}'$ on one subphase. In order for our proof to work, we can pick any *C* with

9 $$\begin{aligned} C>\frac{F+B+\alpha }{\varepsilon } \end{aligned}$$

and any *D* with both

10 $$\begin{aligned} D > r(C + F + B + \alpha ) \end{aligned}$$

and

11 $$\begin{aligned} D > \frac{(1+\varepsilon )r^2C(C+B+F+\alpha )}{r((1+\varepsilon )C-(C+B+F+\alpha ))}\;. \end{aligned}$$

Consider an initial situation *I*, an input sequence $x=(x_1,\dots ,x_n)$, and let the optimal cost of the input (*I*, *x*) be between $(k-1)C$ and *kC* for some integer *k*. Then *x* can be partitioned into *k* phases $\tilde{x}_1=(x_1,\dots ,x_{n_2-1})$, $\tilde{x}_2=(x_{n_2},\dots ,x_{n_3-1}),\dots , \tilde{x}_k=(x_{n_{k}},\dots ,x_n)$ in such a way that $n_i$ is the minimal index for which the optimal cost of the input $(I,(x_1,\dots ,x_{n_i}))$ is at least $(i-1)C$. It follows that the optimal cost for one phase is at least $C-F$ and less than $C+F$, with the exception of the last phase, which may be cheaper. Note that this partition can be generated by the online algorithm itself, i.e., ${\textsc {Rand}}'$ can determine when a next phase starts. This time, there are two reasons for ${\textsc {Rand}}'$ to perform a reset: at the beginning of each phase and after incurring a cost exceeding *D* since the last reset. Hence, ${\textsc {Rand}}'$ starts each phase with a reset, and the processing of each phase is partitioned into a number of subphases each of cost at least *D* (with the exception of the possibly cheaper last subphase) and at most $D+F$.

Now we are going to discuss the cost of ${\textsc {Rand}}'$ on a particular input. Let us fix the input (*I*, *x*) which subsequently also fixes the indices $1=n_1,\dots ,n_k$. Let $S_i$ be a random variable denoting the state of the problem (according to Definition 4) just before processing request $x_i$, and let *W*(*i*, *j*) with $i\le j$ be a random variable denoting the cost of ${\textsc {Rand}}'$ incurred on the request sequence $x_i,\dots ,x_j$. Claim 1 holds in this setting as well.

The overall structure of the proof is as follows. We show in Lemma 6 that the expected cost obtained by ${\textsc {Rand}}'$ during a phase (conditioned on the state in which the phase was entered) is at most $\mu :=r(C + F + B+\alpha )/(1-p)$, where $p:=r(C + F + B+\alpha )/D < 1$. We can then consider random variables $Z_0,\dots ,Z_k$ such that $Z_0 :=k\mu$ and $Z_{i} :=(k-i)\mu + \sum _{j=1}^i \overline{W}_j$ for $i>0$, where $\overline{W}_i$ is the cost of the *i*th phase, clipped from above by some logarithmic bound, i.e., $\overline{W}_i :=\min \{ W({n_i},{n_{i+1}}-1), c\log k\}$, for some suitable constant *c*. We show in Lemma 7 that $Z_0,\dots ,Z_k$ form a bounded supermartingale, and then use the Azuma–Hoeffding inequality to conclude that $Z_k$ is unlikely to be much larger than $Z_0$. By a suitable choice of the free parameters, this implies that $Z_k$ is unlikely to be much larger than the expected cost of ${\textsc {Rand}}$. Finally, we show that w.h.p. $Z_k$ is the cost of ${\textsc {Rand}}'$.

In order to argue about the expected cost of a given phase in Lemma 6, let us first show that a phase is unlikely to have many subphases. For the rest of the proof, let $X_j$ be the random variable denoting the number of subphases of phase *j*.

### Lemma 5

For any *i*, *s*, and any $\delta \in \mathbb {N}$, we have $Pr [X_{i}\ge \delta \mid S_{n_i}=s]\le p^{\delta -1}$.

### Proof

**Fig. 1:**
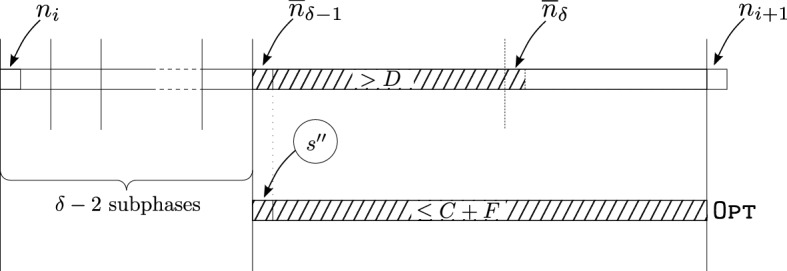
The situation with $\delta$ subphases

The proof is done by induction on $\delta$. For $\delta =1$ the statement holds by definition. Let $\overline{n}_c$ denote the index of the first request after $c-1$ subphases, with $\overline{n}_1={n_i}$, and $\overline{n}_c=\infty$ if there are fewer than *c* subphases. In order to have at least $\delta \ge 2$ subphases, the algorithm must enter some suffix of phase *i* at position $\overline{n}_{\delta -1}$ and incur a cost of more than *D* (see Fig. 1). Hence,

12 $$\begin{aligned}&Pr [X_{i}\ge \delta \mid S_{n_i}=s] = Pr [\overline{n}_{\delta -1}<n_{i+1}-1\mid S_{n_i}=s]\nonumber \\&\qquad \cdot Pr [ W(\overline{n}_{\delta -1},n_{i+1}-1)>D \mid \overline{n}_{\delta -1}<n_{i+1}-1\wedge S_{n_i}=s]\;. \end{aligned}$$

The fact that $\overline{n}_{\delta -1}<n_{i+1}-1$ means that there are at least $\delta -1$ subphases, i.e.,

13 $$\begin{aligned} Pr [\overline{n}_{\delta -1}<n_{i+1}-1\mid S_{n_i}=s] \le Pr [X_{i}\ge \delta -1\mid S_{n_i}=s] \le p^{\delta -2} \end{aligned}$$

by the induction hypothesis. We decompose the event and obtain

14 $$\begin{aligned}&Pr [ W(\overline{n}_{\delta -1}, n_{i+1}-1)>D \mid \overline{n}_{\delta -1}<n_{i+1}-1\wedge S_{n_i}=s] \nonumber \\&\quad = \sum _{\begin{array}{c} i',s' \\ n_i\le i'<n_{i+1}-1 \end{array}} Pr [ W(\overline{n}_{\delta -1}, n_{i+1}-1)>D \mid \overline{n}_{\delta -1}=i'\wedge S_{i'}=s']\nonumber \\&\qquad \cdot Pr [\overline{n}_{\delta -1}=i'\wedge S_{i'}=s' \mid \overline{n}_{\delta -1}<n_{i+1}-1\wedge S_{n_i}=s]\;. \end{aligned}$$

Now let us argue about the probability

$$\begin{aligned} Pr [ W(\overline{n}_{\delta -1}, n_{i+1}-1)>D \mid \overline{n}_{\delta -1}=i'\wedge S_{i'}=s'\wedge S_{n_i}=s]\;. \end{aligned}$$

${\textsc {Rand}}'$ performed a reset just before reading $x_{i'}$, so it starts simulating ${\textsc {Rand}}$ from state $s'$. However, in the optimal solution, there is some state $s''$ associated with position $i'$ such that the cost of the remainder of the *i*th phase is at most $C+F$. Due to the assumption of the theorem, the optimal cost on the input $x_{i'},\dots ,x_{n_{i+1}-1}$ starting from state $s'$ is at most $C+F+B$, and the expected cost incurred by ${\textsc {Rand}}$ is at most $r(C+F+B) + \alpha$. Using Markov’s inequality, we get

15 $$\begin{aligned} Pr [W(\overline{n}_{\delta -1}, n_{i+1}-1)>D \mid \overline{n}_{\delta -1}=i'\wedge S_{i'}=s'] \le \frac{r(C + F + B) + \alpha }{D} \le p\;.\nonumber \\ \end{aligned}$$

Plugging Lemma 15 into Lemma 14, and then together with Lemma 13 into Lemma 12 yields the result. $\square$

Now we can argue about the expected cost of a phase.

### Lemma 6

For any *i* and *s*, it holds that $\mathbb {E} [W(n_i,n_{i+1}-1) \mid S_i=s]\le \mu$.

### Proof

Let $\overline{n}_c$ be defined as in the proof of Lemma 5. Using the same arguments, we have that the expected cost of a single subphase is

$$\begin{aligned} \mathbb {E} [ W(\overline{n}_c,\min \{\overline{n}_{c+1},n_{i+1}-1\}) \mid \overline{n}_c=i'\wedge S_{i'}=s' ] \le r(C+F+B) + \alpha \;. \end{aligned}$$

Conditioning and decomposing by $\overline{n}_c$ and $s'$, we get that

$$\begin{aligned} \mathbb {E} [ W(\overline{n}_c,\min \{\overline{n}_{c+1},n_{i+1}-1\}) \mid X_i\ge c] \le r(C+F+B) + \alpha \;. \end{aligned}$$

Finally, let $Q_{i,c}= W(\overline{n}_c,\min \{\overline{n}_{c+1},n_{i+1}-1\})$ if $X_i\ge c$, or 0 if $X_i<c$. This yields

$$\begin{aligned}&\mathbb {E} [W(n_i, n_{i+1}-1) \mid S_i=s] = \sum _{c=1}^\infty \mathbb {E} [ Q_{i,c} \mid S_i=s] \\&\quad = \sum _{c=1}^\infty \mathbb {E} [ Q_{i,c} \mid S_i=s \wedge X_i\ge c]\cdot Pr [X_i\ge c]\\&\quad \le \sum _{c=1}^\infty (r(C+F+B)+\alpha ) p^{c-1} = (r(C+F+B)+\alpha )/(1-p) \le \mu \;, \end{aligned}$$

which proves the lemma. $\square$

Once the expected cost of a phase is established, we can show that $Z_0,\dots ,Z_k$ form a bounded supermartingale using arguments similar to the proof of Lemma 2.

### Lemma 7

For any constant $c>0$, the sequence $Z_0,\dots ,Z_k$ is a supermartingale.

### Proof

Consider a fixed *c*. This time, we have to show that for each *i*, $\mathbb {E} [Z_{i+1}\mid Z_0,\dots ,Z_i]\le Z_i$. From the definition of the $Z_i$’s it follows that $Z_{i+1}-Z_i=\overline{W}_{i+1}-\mu$. Again, consider any elementary event $\xi$ from the probability space, and let $Z_i(\xi )=z_i$, for $i=0,\dots ,k$, be the values of the corresponding random variables. We have

$$\begin{aligned}&\mathbb {E} [Z_{i+1}\mid Z_0,\dots ,Z_i](\xi ) = \mathbb {E} [ Z_{i+1}\mid Z_0=z_0,\dots ,Z_i=z_i]\\&\quad = \mathbb {E} [ Z_i+\overline{W}_{i+1}-\mu \mid Z_0=z_0,\dots ,Z_i=z_i]\\&\quad = z_i-\mu +\mathbb {E} [\overline{W}_{i+1}\mid Z_0=z_0,\dots ,Z_i=z_i]\\&\quad = z_i-\mu +\sum _s\mathbb {E} [\overline{W}_{i+1}\mid Z_0=z_0, \dots ,Z_i=z_i,S_{n_{i+1}}\!=s]\\&\qquad \cdot Pr [S_{n_{i+1}}\!=s \mid Z_0=z_0,\dots ,Z_i=z_i]\\&\quad \le z_i-\mu +\sum _s\mathbb {E} [W(n_{i+1},n_{i+2}-1)\mid S_{n_{i+1}}\!=s]\cdot Pr [S_{n_{i+1}}\!=s \mid Z_0=z_0,\dots ,Z_i=z_i]\\&\quad \le z_i-\mu +\mu \sum _sPr [S_{n_{i+1}}=s \mid Z_0=z_0,\dots ,Z_i=z_i]=z_i=Z_i(\xi )\;, \end{aligned}$$

where the last inequality is a consequence of Lemma 6. $\square$

Now we use the following special case of the Azuma–Hoeffding inequality [1, 6].

### Lemma 8

(Azuma, Hoeffding) Let $Z_0,Z_1,\dots$ be a supermartingale, such that $|Z_{i+1}-Z_i|<\gamma$. Then for any positive real *t*,

$$\begin{aligned} Pr [ Z_k - Z_0 \ge t]\le \exp \left( -\frac{t^2}{2k\gamma ^2}\right) \;. \end{aligned}$$

In order to apply Lemma 8, we need the following bound.

### Claim 3

Let *k* be such that $c\log k>\mu$. For any *i*, it holds that $|Z_{i+1}-Z_i|<c\log k$.

We are now ready to prove the subsequent lemma.

### Lemma 9

Let *k* be such that $c\log k>\mu$. If *C* and *D* satisfy (9), (10), and (11), then

$$\begin{aligned} Pr [Z_k\ge (1+\varepsilon )rkC]\le \exp \left( -\frac{k\left( (1+\varepsilon )rC -\mu \right) ^2}{2c^2\log ^2k}\right) \;. \end{aligned}$$

### Proof

Applying Lemma 8 for any positive *t*, we get

$$\begin{aligned} Pr [Z_k-Z_0\ge t]\le \exp \left( -\frac{t^2}{2kc^2\log ^2k}\right) \;. \end{aligned}$$

Noting that $Z_0=k\mu$ and choosing

$$\begin{aligned} t:=k((1+\varepsilon )rC-\mu )\;, \end{aligned}$$

the statement follows. The only remaining task is to verify that $t>0$, i.e.,

$$\begin{aligned} (1+\varepsilon )rC>r(C+F+B+\alpha )\frac{1}{1-\frac{r(C+F+B + \alpha )}{D}}\;. \end{aligned}$$

Due to (9), $(1+\varepsilon )C>C+F+B+\alpha$. Furthermore, due to (11), we have

$$\begin{aligned} rD(1+\varepsilon )C-rD(C+B+F+\alpha )>(1+\varepsilon )r^2C(C+B+F+\alpha ) \end{aligned}$$

and therefore

$$\begin{aligned} (1+\varepsilon )rC(D-r(C+B+F+\alpha ))>rD(C+B+F+\alpha )\;, \end{aligned}$$

and the claim follows. $\square$

To get to the statement of the main theorem, we show the following technical bound.

### Lemma 10

For any *c* and $\beta >1$, there is a $k_0$ such that for any $k>k_0$, we have

$$\begin{aligned} \exp \left( -\frac{k\left( (1+\varepsilon )rC-\mu \right) ^2}{2c^2\log ^2k}\right) \le \frac{1}{2(2+kC)^\beta }\;. \end{aligned}$$

### Proof

Note that the left-hand side is of the form $\exp \left( -\eta \frac{k}{\log ^2k}\right)$ for some positive constant $\eta$. Clearly, for any $\beta >1$ and sufficiently large *k*, it holds that $\exp \left( \eta \frac{k}{\log ^2k}\right) \ge 2(2+kC)^\beta$. $\square$

Combining Lemmas 9 and 10, we get the following result.

### Corollary 1

If *C* and *D* satisfy (9), (10) and (11), then for any $\beta >1$ there is a $k_0$ such that for any $k>k_0$, we have

$$\begin{aligned} Pr [Z_k\ge (1+\varepsilon )rkC]\le \frac{1}{2(2+kC)^\beta }\;. \end{aligned}$$

To finish the proof, we show that w.h.p. $Z_k$ is actually the cost of ${\textsc {Rand}}'$.

### Lemma 11

For any $\beta >1$, there is a *c* and a $k_1$ such that, for any $k>k_1$ and any input sequence *x* resulting in *k* phases,

$$\begin{aligned} Pr [Z_k \ne cost ({\textsc {Rand}}' (I,x)) ]\le \frac{1}{2(2+kC)^\beta }\;. \end{aligned}$$

### Proof

Since $Z_k=\sum _{j=1}^k\min \{W(n_j,n_{j+1}-1),c\log k\}$ the event that $Z_k\ne cost ({\textsc {Rand}}' (I,x))$ happens exactly when there is some *j* such that $W(n_j,n_{j+1}-1)>c\log k$.

Consider any fixed *j*. Since the cost of a subphase is at most $D+F$, it holds that $W(n_j,n_{j+1}-1)\le X_j(F+D)$. From Lemma 5, it follows that for any *c* that

$$\begin{aligned} Pr [W(n_j,n_{j+1}-1)>c\log k]\le Pr \left[ X_j\ge \left\lceil \frac{c\log k}{F+D}\right\rceil \right] \le p^{\frac{c\log k}{F+D}-1}\;. \end{aligned}$$

Consider the function

$$\begin{aligned} g(k):=\frac{\log \left( \frac{2k}{p}(2+kC)^\beta \right) }{\log k}\;, \end{aligned}$$

which is decreasing; furthermore, $\lim _{k\rightarrow \infty }g(k)=1+\beta$. Hence, it is possible to find a constant *c* and a $k_1$, such that for any $k>k_1$ it holds that

$$\begin{aligned} c\ge \frac{F+D}{\log \left( \frac{1}{p}\right) }\cdot g(k)\;. \end{aligned}$$

From that it follows that

$$\begin{aligned} \frac{\log \left( \frac{1}{p}\right) c\log k}{F+D}\ge \log \left( \frac{1}{p}\right) +\log \left( 2k(2+kC)^\beta \right) \end{aligned}$$

and

$$\begin{aligned} \log \left( \frac{1}{p}\right) \left( \frac{c\log k}{F+D}-1\right) \ge \log \left( 2k(2+kC)^\beta \right) \;, \end{aligned}$$

i.e.,

$$\begin{aligned} \left( \frac{1}{p}\right) ^{\frac{c\log k}{F+D}-1}\ge 2k(2+kC)^\beta \;. \end{aligned}$$

Therefore, for this choice of *c* and $k_1$, it holds that

$$\begin{aligned} Pr [W(n_j,n_{j+1}-1)>c\log k]\le p^{\frac{c\log k}{F+D}-1}\le \frac{1}{2k(2+kC)^\beta }\;. \end{aligned}$$

Using the union bound, we conclude that the probability that the cost of any phase exceeds $c\log k$ is at most $1/(2(2+kC)^\beta )$. $\square$

Using the union bound once more, combining Lemmas 11 and 1, and noting that the cost of the optimum is at most *kC*, we get the following statement.

### Corollary 2

If *C* and *D* satisfy (9), (10), and (11), then, for any $\beta >1$, there is a $k_2$ such that, for any $k>k_2$ and any input sequence *x* resulting in *k* phases,

$$\begin{aligned} Pr [cost ({\textsc {Rand}}' (I,x)) \ge (1+\varepsilon )\cdot r\cdot cost ({\textsc {Opt}} (I,x)) ]\le \frac{1}{(2+kC)^\beta }\;. \end{aligned}$$

### Proof

(of Theorem 2) To show that, for any $\beta >1$, there is some $\alpha '$ such that

$$\begin{aligned} Pr \left[ cost ({\textsc {Rand}}' (I,x)) >(1+\varepsilon )\cdot r\cdot cost ({\textsc {Opt}} (I,x)) + \alpha '\right] \le \frac{1}{(2+kC)^\beta } \end{aligned}$$

holds for all *k*, we have to choose $\alpha '$ sufficiently large to cover the cases of $k<k_2$. For these cases, $cost ({\textsc {Opt}} (I,x)) <k_2C$, and hence the expected cost of ${\textsc {Rand}}$ is at most $rk_2C+\alpha$, and due to Lemma 6 the expected cost of ${\textsc {Rand}}'$ is constant. The right-hand side $(2+kC)^{-\beta }$ is decreasing in *k*, so it is at least $(2+k_2C)^{-\beta }$, which is again constant. From Markov’s inequality it follows that there is a constant $\alpha '$ such that

$$\begin{aligned} Pr \left[ cost ({\textsc {Rand}}' (I,x)) >\alpha '\right] <\frac{1}{(2+k_2C)^\beta }\;, \end{aligned}$$

finishing the proof. $\square$

## Applications

We now discuss the impact of our results on paging, the *k*-server problem, and task systems. Despite being related, these problems have different flavors when analyzing them in the context of high probability results.

### Paging and the *k*-Server Problem

Paging allows for a direct application of Theorem 2. Thus, for any paging algorithm with expected competitive ratio *r* there is an algorithm with competitive ratio $r(1+\varepsilon )$ w.h.p..

The *k*-server problem, introduced by Manasse et al. [13], is concerned with the movement of *k* servers in a metric space. Each request is a location and the algorithm has to move one of the servers to that location. If the metric space is finite, this problem is well known to be a special metrical task system.

Theorem 3 directly implies that all algorithms with a constant expected competitive ratio for the *k*-server problem in a finite metric space can be transformed into algorithms that have almost the same competitive ratio w.h.p..

If the metric space is infinite, an analogous result is still valid except that we have to bound the maximum transition cost by a constant. This is the case, because the proof of Theorem 3 uses the finiteness of the state space only to ensure bounded transition costs.

### Task Systems

The properties of online problems needed for Theorem 2 are related to the definition of task systems. There are, however, some important differences.

To analyze the relation, recall the definition of task systems as introduced by Borodin et al. [4]. We are given a finite state space *S*, an initial state $s_0\in S$, and a function $d:S \times S \rightarrow \mathbb {R} _+$ that specifies the (finite) cost to move from one state to another; if *d* is a metric function, we speak of a *metrical task system*. The requests given as input to a task system are a sequence of |*S*|-vectors that specify, for each state, the cost to process the current task if the system resides in that state. An online algorithm for task systems aims at finding a schedule such that the overall cost for transitions and processing is minimized. From now on we will call states in *S* *system states* to distinguish them from the states of Definition 4. As a matter of fact, the concept of states introduced in Definition 4 is more general than system states. As states depend on the sequence of requests and answers, there may be infinitely many states for some online problems. Also, specific state transitions may be impossible. For task systems, however, the states of Definition 4 are the same as system states.

#### Theorem 3

Let ${\textsc {Rand}}$ be a randomized online algorithm with expected competitive ratio *r* for task systems. Then, for any $\varepsilon > 0$, there is a randomized online algorithm ${\textsc {Rand}}'$ for task systems with competitive ratio $(1+\varepsilon )r$ w.h.p..

#### Proof

Consider any task system problem with a fixed set of system states and distance metric. Since ${\textsc {Rand}}$ is a general algorithm for task systems, it can solve the considered task system problem regardless of the choice of the initial state. For the remainder of the proof, we consider a generalized variant of the fixed task system problem where the initial state is part of the input instance and ${\textsc {Rand}}$ to be an algorithm solving this generalized problem.

The task system problem clearly has a partition function according to Definition 3 as each request is associated with a non-negative cost. The adversary may also stop after an arbitrary request, which is already sufficient to induce a unique partition function.

The system states are exactly the states according to our definition, because the optimal future cost only depends on the current system state and a future request has the freedom to assign individual costs to each of the system states. In other words, an equivalence class *s* from Definition 4 (i.e., one state) consists of exactly one unique system state. To apply Theorem 2, we choose the constant *B* of the theorem to be $\max _{s,t \in S}\{d(s,t)\}$. This way, the problem is ${\textsc {Opt}}$-bounded as one transition of cost at most *B* is sufficient to move to any system state used by an optimal algorithm.

The remaining condition of Theorem 2, namely that every state is initial, is satisfied, since we generalized ${\textsc {Rand}}$ to be an algorithm to handle arbitrary initial states. Thus we can apply Theorem 2 to ${\textsc {Rand}}$ and the claim follows. $\square$
